# Fractionally charged skyrmions in fractional quantum Hall effect

**DOI:** 10.1038/ncomms9981

**Published:** 2015-11-26

**Authors:** Ajit C. Balram, U. Wurstbauer, A. Wójs, A. Pinczuk, J. K. Jain

**Affiliations:** 1 Department of Physics, 104 Davey Lab, Pennsylvania State University, University Park, Pennsylvania 16802, USA; 2 Walter Schottky Institut and Physik-Department, Am Coulombwall 4a, Technische Universität München, D-85748 Garching, Germany; 3 Nanosystems Initiative Munich (NIM), Schellingstraße 4, 80799 München, Germany; 4 Department of Theoretical Physics, Wrocław University of Technology, 50-370 Wrocław, Poland; 5 Department of Applied Physics and Applied Mathematics and Department of Physics, Columbia University, New York 10027, USA

## Abstract

The fractional quantum Hall effect has inspired searches for exotic emergent topological particles, such as fractionally charged excitations, composite fermions, abelian and nonabelian anyons and Majorana fermions. Fractionally charged skyrmions, which support both topological charge and topological vortex-like spin structure, have also been predicted to occur in the vicinity of 1/3 filling of the lowest Landau level. The fractional skyrmions, however, are anticipated to be exceedingly fragile, suppressed by very small Zeeman energies. Here we show that, slightly away from 1/3 filling, the smallest manifestations of the fractional skyrmion exist in the excitation spectrum for a broad range of Zeeman energies, and appear in resonant inelastic light scattering experiments as well-defined resonances slightly below the long wavelength spin wave mode. The spectroscopy of these exotic bound states serves as a sensitive tool for investigating the residual interaction between composite fermions, responsible for delicate new fractional quantum Hall states in this filling factor region.

## Abstract (web summary)

It is predicted that fractionally charged skyrmions, topologically protected vortex-like spin configurations, may exist in systems exhibiting fractional quantum Hall states. Here, the authors demonstrate the existence of such objects in GaAs single quantum wells.

## Graphical abstract

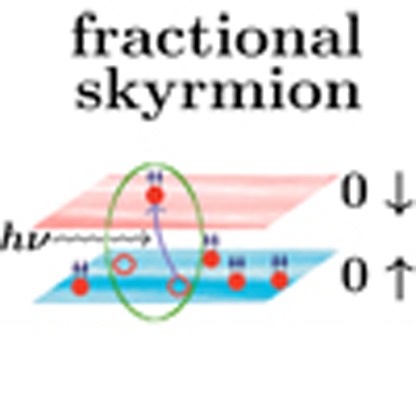

Skyrmions represent vortex-like spin structures in two dimensions, which are two-dimensional stereographic projections of the spin hedgehog on a sphere. In a pioneering work, Sondhi *et al*.^1^ predicted integrally charged skyrmions for the quantum Hall ferromagnet near filling factor *ν*=1. These arise because the *ν*=1 integer quantum Hall effect state exhibits spontaneous ferromagnetism even in the absence of a Zeeman energy with the remarkable property that the addition or removal of a single electron causes a macroscopic number of spin flips^1^. For non-zero Zeeman energies, the number of spin flips depends on the competition between the exchange and the Zeeman energies^2^, that is, on the parameter 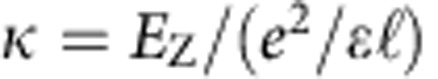, which characterizes the strength of the Zeeman splitting *E*_Z_=*gμ*_B_*B*. (Here *ɛ* is the dielectric constant of the background material, *B* is the perpendicular magnetic field, 
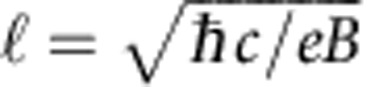
 is the magnetic length, *g* is the Landé g-factor, and *μ*_B_ is the Bohr magneton. For the parameters of GaAs with the magnetic field specified in units of Tesla (T), we have 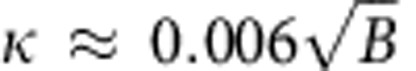.) The skyrmion physics is relevant for *κ*≲0.05 (*B*≲70 T for GaAs), and has been confirmed experimentally^3^,^4^.

The fractional quantum Hall effect^5^ (FQHE) arises due to the formation of composite fermions, which are topological bound states of electrons and an even number (2*p*) of quantized vortices^6^. Composite fermions experience an effective magnetic field and form Landau-like levels called Λ levels (ΛLs). Their filling factor ν* is related to the electron filling factor *ν* by the expression *ν*=ν*/(2pν*±1). The FQHE states at *ν*=*n*/(2*n*±1) are manifestations of integer quantum Hall effect of composite fermions and one may expect fractionally charged skyrmions close to composite fermion filling, ν*=1, which corresponds to the *ν*=1/3 state^7^. Such fractional skyrmions (FSs) were already predicted in the work of Sondhi *et al*.^1^, and their existence was subsequently verified in detailed microscopic calculations^8^,^9^,^10^. These calculations indicated, however, that the fractional skyrmions are much more delicate than the integral skyrmions near *ν*=1, because the exchange interaction between composite fermions is much weaker than that between electrons. It was estimated that fractional skyrmions should occur only below *κ*≈0.009 (*B*≲2.5 T for GaAs). Consequently, for typical experimental parameters, when the filling factor is varied away from ν*=1 (or from *ν*=1/3), trivial quasiparticles, namely isolated composite fermion particles or holes, are produced rather than fractional skyrmions. The fractional skyrmions have been probed experimentally by suppressing *g* through the application of hydrostatic pressure^11^. Certain *E*_Z_ dependencies of the excitations^12^,^13^ at *ν*=1/3 have also been interpreted in terms of skyrmion physics, but it is unclear how skyrmions may occur at the high *κ* values of these experiments, and an alternative explanation of the observations has been proposed^14^. The binding energy of fractional skyrmions has not been measured so far, which would be important for a convincing observation.

This work is concerned with minimal fractional skyrmions, namely the skyrmions for which a composite fermion (CF) particle or hole is dressed by a single additional spin-flip exciton (SFE). We show theoretically that such skyrmions exist in the excitation spectrum just below the Zeeman energy for a broad range of *κ* at filling factors slightly away from *ν*=1/3. These are accessible in resonant inelastic light scattering (RILS), because a photoexcited SFE that can bind, for *ν*>1/3 (*ν*<1/3), with a pre-existing composite fermion particle (hole) to produce a negatively (positively) charged fractional skyrmion, denoted by FS^−^ (FS^+^). We identify certain modes observed in RILS experiments^15^,^16^ with the minimal fractional skyrmions, supporting this identification by a detailed analysis of the experimental data, which shows qualitative and quantitative agreement between theory and experiment. In particular, the measured binding energies of the positively and negatively charged fractional skyrmions are seen to be in excellent agreement with the calculated binding energies.

## Results

### Theory

**Figure 1:**
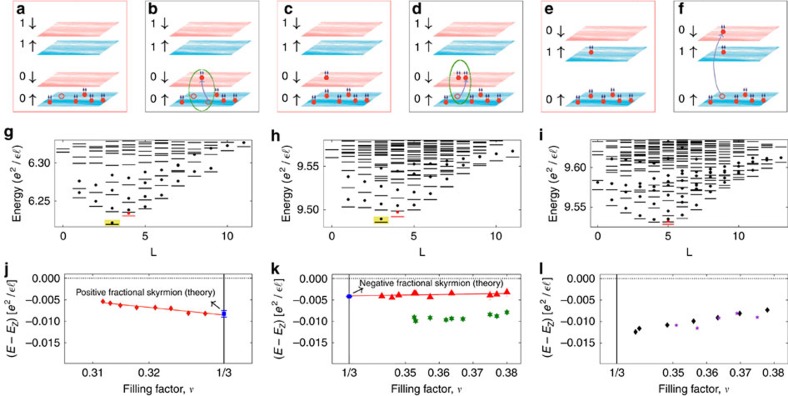
Comparison between theory and experiment. (**a**) shows the ground state for the fully polarized state for *v*≲1/3 with a single composite fermion (CF) hole (empty red circle) in the spin-up lowest ΛL (0↑); (**b**) shows an additional spin-flip exciton (SFE) that binds with the hole to produce a minimal positively charged fractional skyrmion (FS). (**c**) shows the state for *v*≳1/3 with a single CF particle in the spin-down lowest ΛL (0↓), and (**d**) has an additional SFE. (**e**) has a CF particle in the spin-up second ΛL (1↑), and (**f**) has an additional SFE. The composite fermions are shown as particles with two arrows, representing bound vortices, and their up and down spin ΛLs are shown as shaded blue and red rectangles, respectively. In **g**–**i** the red dashes (dots) show the exact (CF) energies of the ground states containing a single CF particle or hole (as shown in **a**,**c**,**e**) and the black symbols show the spectrum obtained when an additional SFE is created (as shown in **b**,**d**,**f**). The spherical geometry is used for calculations; panel (**g**) is for eight particles subjected to 22 flux quanta (a flux quantum is defined as *φ*_0_=*hc*/*e*), and (**h**,**i**) correspond to 10 particles in 26 flux quanta. (**j**–**l**) show the experimentally measured energies of modes below the Zeeman energy. The theoretical energy of the FSs in the dilute limit of *ν*→1/3 including finite width correction is also shown by blue square. Panels (**j**,**l**) are for 50° tilt, whereas (**k**) is for 30° tilt. All energies in **j**–**l** are shown relative to the Zeeman energy, in units of 
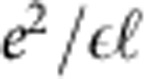
, where 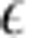 is the dielectric constant of the material and 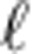 is the magnetic length. The modes depicted by red symbols are assigned to fractional skyrmions, green stars in panel **k** to the excitation shown in Fig. 8d, and the black diamonds and purple stars in panel **l** to the excitation shown in Fig. 8i. The theoretical error bars arise from the uncertainty in the Monte Carlo calculations and thermodynamic extrapolations, and the experimental error bar reflects the uncertainty in the Lorentzian fits.

Employing a combination of exact and composite fermion diagonalization methods, we evaluate the binding energy of the minimal fractional skyrmion, that is, the amount by which it lies below the Zeeman energy, and estimate corrections due to finite quantum well thickness. We consider filling factors close to *ν*=1/3, where the density of composite fermion particles or holes is dilute and it suffices to consider a single composite fermion particle or hole. The composite fermion hole resides in the spin-up lowest ΛL (0↑ ΛL) for all *κ* (Fig. 1a), whereas the composite fermion particle can reside either in spin-down lowest ΛL (0↓ ΛL) for small *κ* (Fig. 1c) producing a partially polarized state, or in spin-up second ΛL (1↑ ΛL) for large *κ* (Fig. 1e) producing a fully spin-polarized state.

We use the spherical geometry^17^, in which *N* electrons move on the surface of a sphere, exposed to a radial magnetic field that produces a flux of 2*Qφ*_0_ through the surface of the sphere, where 2*Q* is a positive integer and *φ*_0_=*hc*/*e* is a unit flux quantum. The distance between the electrons is defined as the chord distance on the sphere; whether the chord or the arc distance is chosen is unimportant because we evaluate the thermodynamic limit of the energy. The *ν*=1/3 state occurs at 2*Q*=3*N*−3 and has the spin quantum number *S*=*N*/2. A single composite fermion hole occurs at 2*Q*=3*N*−2 with the spin of the bare CF particle (Fig. 1a) also given by *S*=*N*/2. The composite fermion particle at 2*Q*=3*N*−4 can go either into 0↓ ΛL (Fig. 1c), with spin *S*=*N*/2−1, or into 1↑ ΛL (Fig. 1e), with spin *S*=*N*/2. The red dashes in Fig. 1g–i are obtained by exact diagonalization in these spin sectors. We can also construct explicit wave functions for these states in the composite fermion theory, which for the CF hole has the form 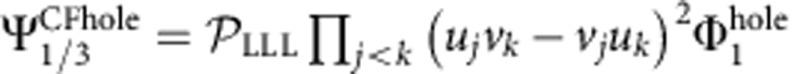 where 
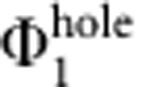
 is the known wave function of a single hole at *ν*=1, *u*=cos(*θ*/2)exp(*iφ*/2) and *v*=sin(*θ*/2)exp(−*iφ*/2) are spinor coordinates, *θ* and *φ* are the polar and azimuthal angles on the sphere, and 
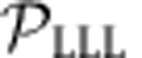
 represents projection into the lowest Landau level (LLL). Wave functions for spin-conserving and spin-reversed composite fermion particles are constructed analogously. The red dots in g, h and i of Fig. 1 are the Coulomb energies of these wave functions.

To consider the fractional skyrmions, we next consider states containing an additional SFE, shown in Fig. 1b,d,f and consider the sector Δ*S*=−1, where Δ*S* is measured relative to the ground state. We show the spectra in these spin sectors in Fig. 1 in g, h and i. The black dashes show the exact Coulomb spectra obtained by numerical diagonalization. The black dots are the spectra obtained by the method of composite fermion diagonalization^18^. For the latter, we first construct a basis of all states in the relevant spin sector at 2Q*=2*Q*−2(*N*−1) (which is the effective flux experienced by composite fermions), denoted by 
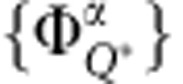
, where α labels different basis functions. We then composite fermionize this basis to obtain the correlated composite fermion basis at 2*Q*, given by 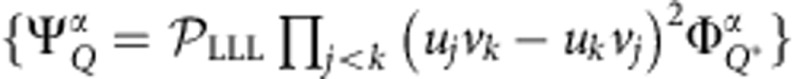. We finally diagonalize the Coulomb interaction in this basis to obtain eigen energies and eigen functions.

**Figure 2:**
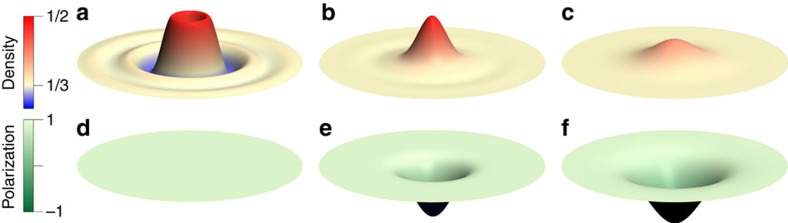
Contrasting negatively charged skyrmion with composite fermion (CF) particle. (**a**–**c**) show charge density profiles of a spin-conserving CF particle, a spin-reversed CF particle, and a negatively charged fractional skyrmion. Their spin polarization, defined by 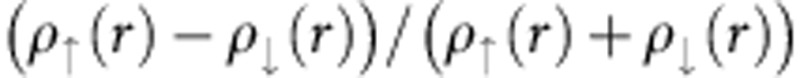 where 
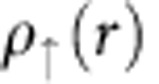
 and 
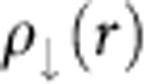
 are the spatial densities of spin-up and spin-down composite fermions, is shown in **d**–**f**, respectively. The minimum/maximum values of the colour bars in each panel are: (**a**) 0.303/0.453, (**b**) 0.333/0.456, (**c**) 0.333/0.391, (**d**) 1.000/1.000, (**e**) −0.352/1.000, (**f**) −0.512/1.000. The disk has a radius of 12.5 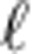.

**Figure 3:**
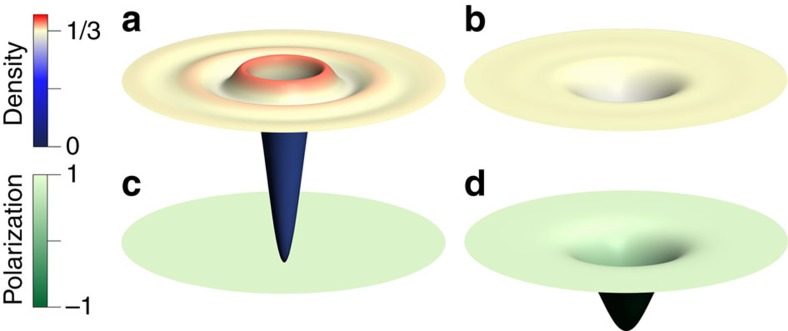
Contrasting the positively charged skyrmion with the composite fermion (CF) hole. (**a**,**b**) show charge density profiles of a CF hole and a positively charged fractional skyrmion. Their spin polarization, defined by 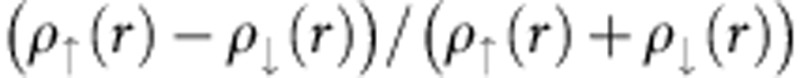 where 
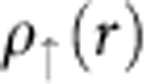
 and 
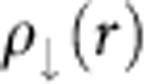
 are the spatial densities of spin-up and spin-down composite fermions, is shown in **c**,**d**, respectively. The minimum/maximum values of the colour bars in each panel are: (**a**) 0.006/0.357, (**b**) 0.266/0.333, (**c**) 1.000/1.000, (**d**) −0.695/1.000. The disk shown has a radius of 12.5 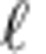.

**Figure 4:**
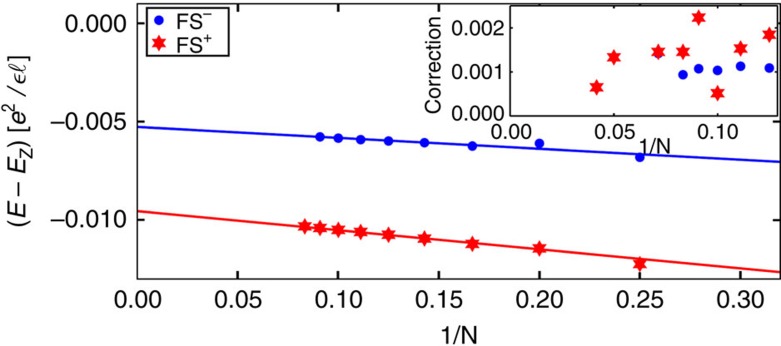
Thermodynamic extrapolation of the binding energies of the fractional skyrmions. The blue (red) symbols show the energies of negative (positive) fractional skyrmions for a system of *N* particles with zero transverse width, obtained from exact diagonalization. The inset shows the amount by which finite-width corrections lower the energy of the fractional skyrmion (FS) for a sample of width 33 nm.

In both the exact and the composite fermion spectra, we find that a fractional skyrmion bound state (highlighted in yellow in panels Fig. 1g,h) is produced when a composite fermion particle in the 0↓ ΛL or a composite fermion hole in the 0↑ ΛL is dressed by the SFE. No such bound state is produced for the fully spin polarized state at *ν*>1/3, that is, for a composite fermion particle in the 1↑ ΛL. The exact density profiles of the fractional skyrmions are seen to be qualitatively different, and much smoother, than those of the composite fermion particle or composite fermion hole (Figs 2 and 3), which is what results in the lowering of the Coulomb energy. Despite the remarkably different structures, they all carry a precise fractional charge of magnitude *e*/3. Figure 4 shows the thermodynamic extrapolation of the binding energy of the fractional skyrmions, denoted 
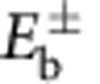
, obtained from exact diagonalization results for finite systems. (The energy of FS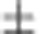 is given by 
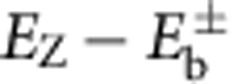
.) The thermodynamic limits for the binding energies are determined to be 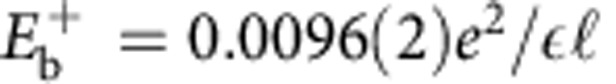 and 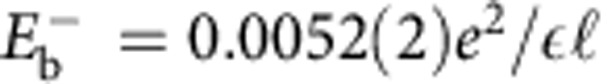 for a system with zero thickness and no Landau level mixing.

**Figure 5:**
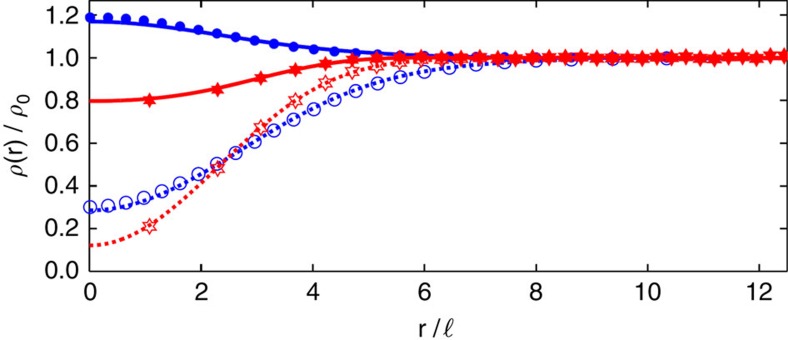
Comparison of exact and composite fermion (CF) density profiles for fractional skyrmions. This figure shows the total density (*ρ*) and the density of spin-up particles 
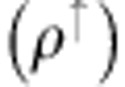
 for fractional skyrmion (FS^−^) (blue) and FS^+^ (red) obtained from exact (dotted and dashed lines) and CF diagonalization (filled and empty symbols). A near perfect overlay of the CF and exact curves shows that the wave function of the FS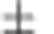 obtained from CF diagonalization is almost identical to the exact one. The results are for 12 particles, and the density is quoted in units of the density of the uniform 1/3 state, denoted by *ρ*_0_.

The interpretation of the fractional skyrmions as bound states of three composite fermions (see panels b and d of Fig. 1) is confirmed by: the close agreement between the energies of the exact and the composite fermion wave functions (that is, the dashes and the dots in Fig. 1); by a comparison of the density profiles of the exact and composite fermion wave functions shown in Fig. 5; and the high overlap of ∼0.99 between the exact and the composite fermion wave functions for *N*=12.

For an accurate quantitative comparison with the experiment, we have estimated corrections due to finite transverse width of the quantum well wave function. We first use a local density approximation^19^ to obtain the transverse wave function *ξ*(*z*). The effective two-dimensional interaction is given by: 
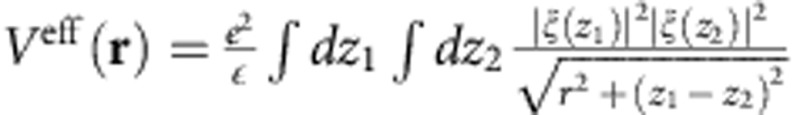
, where *z*_1_ and *z*_2_ are the coordinates perpendicular to the plane containing the electrons. At short distances this interaction is softer than the Coulomb interaction. For the fractional skyrmions, the change in the energy due to finite width is shown in the inset of Fig. 4. We use the composite fermion theory for obtaining the corrections due to finite width, because it is possible to go to larger systems in CFD than in exact diagonalization; the use of the composite fermion theory is justified given the above result showing the accuracy of the composite fermion theory. The finite size variations preclude a clean extrapolation to the thermodynamic limit 1/*N*→0, but it is clear that the binding energies for the fractional skyrmions are reduced only by a small amount. We take the average of all points in the inset of Fig. 4 as a measure of the reduction in the fractional skyrmion binding energy due to finite width, which gives for the positively and negatively charged fractional skyrmions energy reductions of 0.0013±0.0005 and 0.0010±0.0001 
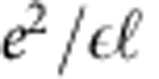
, respectively (with the error given by the standard deviation). We apply this correction to the binding energies obtained from exact diagonalization.

### Experiment

**Figure 6:**
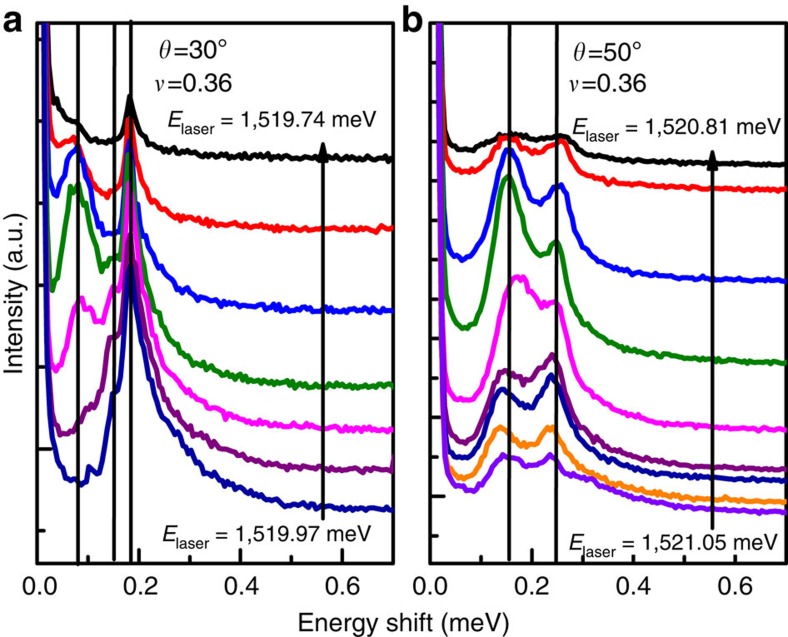
Excitations in resonant inelastic light scattering spectra. (**a**,**b**) show typical spectra at *ν*=0.36 for *θ*=30° and *θ*=50°, respectively, as a function of the incident laser energy *E*_laser_.

**Figure 7:**
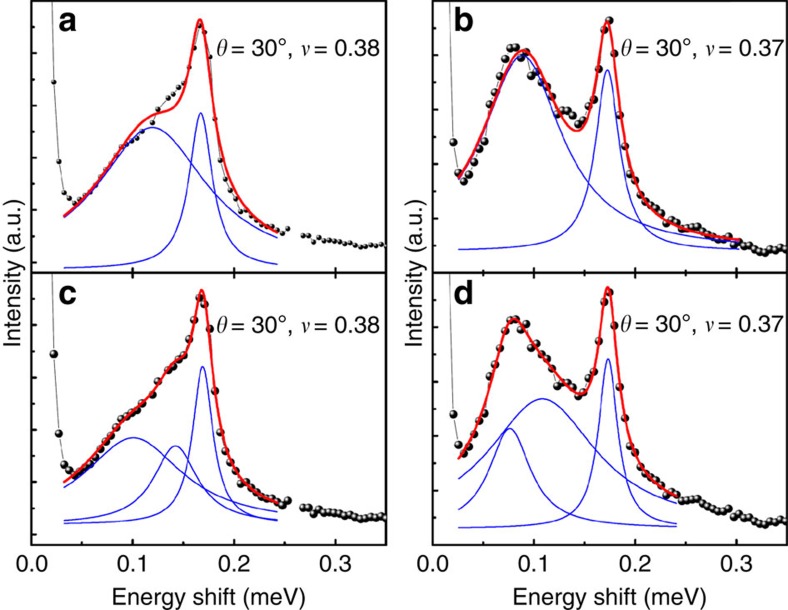
Lorentzian fits to the RILS spectra. Resonant inelastic light scattering (RILS) spectra obtained at 30° tilt for *ν*=0.38 (**a**,**c**) and *ν*=0.37 (**b**,**d**). The raw RILS data are displayed as black bullets and the Lorentzian fits to the data as red solid line. The blue lines in each panel show the individual Lorentzians used to obtain the fit to the data. At 30° tilt the data do not fit well to two Lorentzians as seen in (**a**,**b**) but fit well to three Lorentzians (**c**,**d**).

The experimental results presented in this work are from RILS on a high-quality GaAs single quantum well of width 33 nm, electron density *n*=5.5 × 10^10^ cm^−2^ and low-temperature mobility *μ*=7.2 × 10^6^ cm^2^ Vs^−1^. The magnetic field perpendicular to the sample is *B*=*B*_Total_cos*θ*, where *θ* is the tilt of the sample with respect to the direction of the total magnetic field *B*_Total_. The filling factor and magnetic length depend on the perpendicular field *B*, whereas the Zeeman energy on the total field *B*_Total_, and thus tilting can be used to vary the parameter *κ* (*κ* is defined as the ratio of the Zeeman to Coulomb energy). Measurements were taken at two tilt angles, *θ*=30°±2° and *θ*=50°±2°, which correspond to *κ*≈0.018 and *κ*≈0.023. RILS spectra were obtained by tuning the incident laser photon energy *E*_laser_ to be close to the fundamental optical gap of GaAs to enhance the light-scattering cross-section. To identify all modes, it is important to scan over a range of energies of the incoming laser photon, because modes are picked out by resonant Raman scattering most prominently in a narrow range of parameters where the resonance condition is best satisfied. As seen in Fig. 6a,b, three modes can be identified for 30° tilt, whereas only two are seen for 50° tilt. In many cases, the number of modes and their energies are evident without fitting (see, for example, Fig. 2 in ref. 15 and Fig. 3 in ref. 16). In general, a detailed line shape analysis is necessary to determine the number and the energies of the observed modes. To this end, we determine the least number of Lorentzians that provide a reasonably good fit to the observed Raman line shapes, with the centres of Lorentzians giving the energies of the modes. Some representative fits for 30° tilt are shown in Fig. 7, where at least three Lorentzians are needed for a good fit to the observed line shape. For 50° tilt, a fit with two Lorentzians is found to be satisfactory.

Figure 1g–l show energies of experimental modes observed in RILS at two tilt angles between magnetic field direction and the plane-normal. For 50° tilt the results are taken from Gallais *et al*.^15^. For 30° tilt, the experimental points shown in Fig. 1k are deduced from the RILS spectra of Dujovne *et al*.^16^, but the detailed line shape analysis performed here gives more accurate energies than those quoted in that work.

## Discussion

We identify the mode just below the Zeeman energy with the minimal fractional skyrmion. This identification is supported by several observations. At exactly *ν*=1/3, no modes are observed below *E*_Z_, as expected. For *v*≲1/3, a single sub-*E*_Z_ mode is observed^15^. The excellent quantitative agreement between theory and experiment seen in Fig. 1j confirms its identification with the positively charged fractional skyrmion. We next consider *v*≳1/3. At 30° tilt, the energy of the mode slightly below *E*_Z_ is in excellent agreement with the calculated energy of the the negatively charged fractional skyrmion (including finite thickness correction). We attribute the absence of this mode at 50° tilt to transition from a partially polarized ground state into a fully spin-polarized ground state as *κ* is raised from 0.018 to 0.023 by increasing the tilt. This is consistent with calculations^20^ that have shown that the ground state at *v*≳1/3 has a transition from partially spin-polarized state to a fully spin-polarized state at *κ*≈0.020. We note that unlike their integral counterparts, the positively and negatively charged fractional skyrmions are not related by particle-hole symmetry, as indicated by their different binding energies.

A discussion of various approximations is in order. Landau level mixing and disorder, not included in our calculations, are likely to provide small corrections. Calculations^21^ have shown that Landau level mixing is a minor effect, especially because finite width weakens the short range part of the interaction that is primarily responsible for causing admixture with higher Landau levels. Disorder is also known significantly to diminish the energy of a charged excitation. However, we are concerned here with the change in the energy of a charge-neutral SFE due to its binding with an already present composite fermion particle or hole, which we expect to be less sensitive to disorder^21^. We have also neglected interaction between the fractional skyrmion and other composite fermion particles or holes, which is valid only close to *ν*=1/3 where the density of composite fermion particles or holes is very small. The *ν* dependence of the energy of fractional skyrmion indicates its renormalization due to the presence of other composite fermion particles or holes in its vicinity. The much weaker dependence of the measured dispersion of negatively charged fractional skyrmion indicates a weaker interaction between composite fermion particles. The *ν* dependence of the fractional skyrmion energy can in principle allow a further investigation into the inter-composite fermion interactions, although we have not pursued that here.

The excitation spectrum also contains skyrmions binding *K*>2 SFEs, with energy 
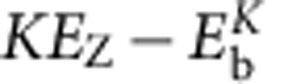
. These lie in the continuum above *E*_Z_ for experimental parameters considered here. We expect that these couple weakly to RILS because they require the incident photon to excite *K*≥2 SFEs, a higher order scattering process.

**Figure 8:**
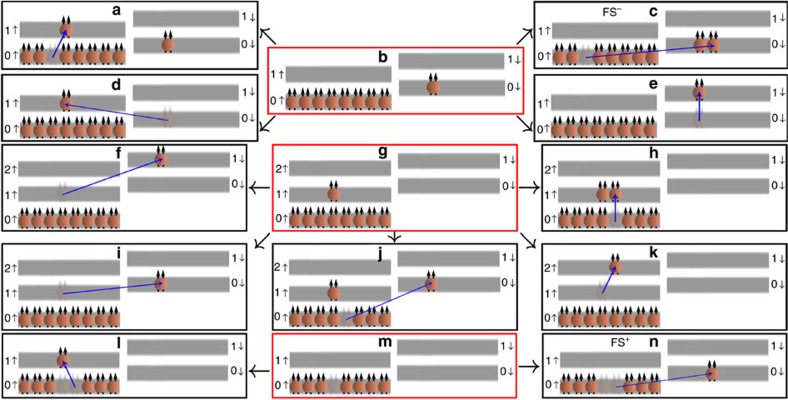
Elementary excitations in the vicinity of filling factor *ν*=1/3. (**b**,**g**) indicate the partially spin-polarized and the fully spin-polarized ground states at *ν*≳1/3, and (**m**) indicates the fully spin-polarized ground state for *ν*≲1/3. The other panels (**a**, **c**–**f**, **h**–**l**, **n**) show the excitations obtained by either promoting (or demoting) a single composite fermion to a higher (lower) Λ level or flipping its spin or doing both. (**c**) shows the negatively charged fractional skyrmion (FS^−^) excitation and (**n**) shows the FS^+^ excitation.

For completeness, we have considered excitations other than the fractional skyrmions. Figure 8 shows schematically various elementary excitations of the states corresponding to a, c and e of Fig. 1. We have performed an exhaustive study of all of these excitations by the standard methods of the composite fermion theory^21^,^22^,^23^, as well as from an extrapolation of the results available from exact diagonalization. The Coulomb contributions to their energy are given in Table 1 for both zero width and the width of 33 nm (which is close to the quantum well width in the experiment). The mode shown in Fig. 8a can be viewed as the mode in Fig. 8d plus a spin wave; given that the Coulomb energy of the spin wave goes to zero at small wave vector according to Larmor's theorem, these two have the same Coulomb energies (although they have different Zeeman contributions). For the modes shown in Fig. 8c,f,i,j (Fig. 8d) we need to add (subtract) the *E*_Z_ before comparing with the experiments.

For *ν*<1/3 only a single sub-*E*_Z_ mode (the positively charged fractional skyrmion) is expected from theory and only one such mode is observed experimentally. For *ν*>1/3 other sub-*E*_Z_ modes are possible. In this region, using the parameters of ref. 16, in addition to the fractional skyrmion, the mode shown in Fig. 8d lies below *E*_Z_ for the partially spin-polarized state, and the mode shown in Fig. 8i lies below the *E*_Z_ for the fully spin-polarized state. We assign the mode in panel l of Fig. 1 to the excitation shown in Fig. 8i. The Coulomb energy contribution to the measured energy, ∼−0.013 
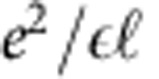
, is to be compared with the theoretical Coulomb energy (including finite-width correction) of −0.018 
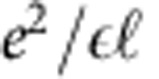
. We further assign the mode in panel k of Fig. 1 indicated by green stars to the excitation shown in Fig. 8d. The Coulomb contribution to the energy of this mode is theoretically calculated to be 0.018 
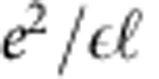
, whereas the measured one is 0.030 
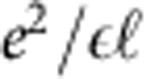
. We find this level of agreement acceptable, considering that both these numbers are differences between the energies of two single particle excitations, which are known to be sensitive to the effects of disorder.

RILS can also detect magnetoroton modes, which are particle-hole excitations of composite fermions (to be distinguished from the minimal fractional skyrmions that are bound state of three composite fermions). The magnetoroton modes of the 1/3 or 2/5 states occur at energies much higher than *E*_Z_ for the parameters of the experiments in question. Additional low energy magnetorotons are in principle possible at incompressible FQHE states of composite fermions in the range 1/3<*ν*<2/5, such as 4/11, 5/13 and 3/8 (refs 24, 25, 26), which correspond to neutral spin-conserving modes of the composite fermions in the minority spin sector^27^,^28^,^29^. Several observations indicate that this physics is not relevant to the mode identified above as the negatively charged fractional skyrmion. First, the composite fermion-FQHE states at 4/11, 5/13 and 3/8 are stabilized at temperatures below the minimum temperature (50 mK) of the experiments of Fig. 1. Second, the experimental mode identified as the fractional skyrmion does not require a fine tuning of *ν*. Third, the energy of the observed mode agrees with the theoretically calculated energy of the fractional skyrmion. Finally, the neutral magnetoroton modes of 4/11, 3/8 and 5/13 are expected to occur at ∼0.002 
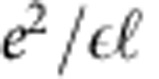
 (refs 30, 31), which is much lower than the energy of the fractional skyrmion.

In conclusion, we have shown that the smallest version of the fractionally charged skyrmions can be created optically in the vicinity of *ν*=1/3 when a photoexcited neutral exciton forms a bound state with an already present charged composite fermion particle or hole. Furthermore, we provide strong evidence that the modes observed slightly below the long-wavelength spin-wave mode at the Zeeman energy are precisely these skyrmions. In particular, the measured binding energy of these skyrmions is in excellent agreement with the theory. The study of skyrmions for *ν*≈1/3 provides a sensitive probe into the inter-composite fermion interaction, and also sheds light on the spin polarization of the ground state.

## Additional information

**How to cite this article:** Balram, A. C. *et al*. Fractionally charged skyrmions in fractional quantum Hall effect. *Nat. Commun.* 6:8981 doi: 10.1038/ncomms9981 (2015).

## Figures and Tables

**Table 1:** Energy of the elementary excitations in the vicinity of *ν*=1/3 shown in Fig. 8.

**Mode**	**Width,** ***w*****=0**		***E***_***w*****=33 nm**_**−*****E***_***w*****=0**_	
	**Exact**	**CF theory**	**CF theory**	
(a)+(e),(d)	0.0284 (3)	0.0258 (0)	−0.0073 (0)
(c)	−0.0052 (2)	∼−0.0059 (7)	0.0010 (0)
(f)+(j)	0	0	0
(h)	0.0422 (33)	∼0.0436 (113)	−0.0095 (207)
(i)	−0.0284 (3)	−0.0258 (0)	0.0073 (0)
(k)	—	0.0867 (1)	−0.0224 (1)
(l)	0.0369 (17)	0.0366 (47)	−0.0117 (77)
(n)	−0.0096 (2)	∼−0.0108 (24)	0.0013 (7)

The Coulomb energy of the the elementary excitations near *ν*=1/3 determined by an extrapolation of the finite system results, obtained by exact diagonalization (second column) and the CF theory (third column), for quantum well width *w*=0. The last column gives the difference in the energies of each mode for quantum wells of widths *w*=33 nm and *w*=0, obtained by the CF theory. All energies are quoted in units of 
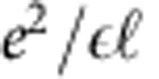
. The cases where linear extrapolation in 1/*N* to the thermodynamic limit is not very accurate are marked by the symbol ∼ to indicate larger uncertainty. The total energy of Fig. 8c, i and n (Fig. 8d) is obtained by adding (subtracting) the Zeeman splitting *E*_Z_ as explained in the text. (Note: A combination of Fig. 8a and e is needed to obtain *S*^2^ eigen states; the same is true of f and j. k is an eigen state of the Hamiltonian but it is in general an excited state.)
